# Who discovers the firearm suicide decedent: an epidemiologic characterization of survivor-victims

**DOI:** 10.1186/s40621-022-00408-3

**Published:** 2022-12-12

**Authors:** Leslie M. Barnard, Colton Leavitt, Talia L. Spark, Jacob B. Leary, Erik A. Wallace

**Affiliations:** 1 Department of Epidemiology, Colorado School of Public Health, Aurora, CO USA; 2 Department of Emergency Medicine, University of Colorado School of Medicine, Aurora, CO USA; 3 University of Utah School of Medicine, Salt Lake City, UT USA; 4 Rocky Mountain MIRECC for Suicide Prevention, U.S. Department of Veterans Affairs, Aurora, CO USA; 5 University of Colorado School of Medicine, Aurora, CO USA; 6 University of Colorado School of Medicine, Colorado Springs, CO USA

**Keywords:** Firearm, Suicide, Suicide survivors, Epidemiology

## Abstract

**Background:**

Suicide is the tenth leading cause of death in the United States, with over half of cases involving firearms. Despite research indicating negative effects of exposure to suicide, there is little research on who typically finds the body of the suicide decedent. Understanding who finds the body of the suicide decedent may be important to understand trauma and mental health effects.

**Findings:**

Of the 332 people who died by suicide in El Paso County, Colorado, 182 (55%) used firearms. Those who died by firearm suicide were more likely to be male (83.5% vs. 67.3%) have military affiliation (39.0% vs. 19.3%) and were less likely to have a known mental health diagnosis (47.3% vs. 64.7%) compared to those who died from other means. Most suicide decedents were found by a family member or friend (60.2%). The remaining decedents were found by a stranger/acquaintance (21.0%) or a first responder (22.4%) One-fifth of suicides involved forced witnessing (19%) and the majority were already deceased when the body was discovered (73.2%).

**Conclusions:**

While most suicide decedents are discovered by a family member or a friend, it is unknown what the bereavement and trauma-related outcomes are among people who discover a suicide decedent who has died by violent means, especially by firearms. Further studies exploring who discovers suicide decedents and targeted postvention strategies for supporting impacted family, friends, first responders, and strangers are needed.

## Background

Suicide is the tenth leading cause of death in the United States, with over half of cases involving firearms (WISQARS 2020). Survivors who discover the body of a suicide decedent or witness the suicide may be at increased risk of mental health diagnoses and suicide from trauma in addition to grief (Tal Young et al. 2012; Pitman et al. 2014). First responders and health care professionals are also at increased risk of suicide themselves due to their occupational exposure to suicide (Lyra et al. 2021). Despite the well-documented deleterious effects of suicide exposure, there is a paucity of data to indicate who typically finds the body of the suicide decedent.

## Objective

To describe (1) differences between firearm and non-firearm suicides and (2) circumstances regarding who discovered the body of firearm and non-firearm suicide decedents.

## Methods and findings

Data on all suicide decedents in 2018–2019 were abstracted from the El Paso County Coroner’s Office, including police and coroner reports. Authors (LB, CL, JL, EW) abstracted data detailing who discovered the decedent, circumstances of the scene and known suicide risk factors. A 15% random sample of firearm decedents (*n* = 28) was double-coded with an inter-rater reliability of 94.0%. Analyses were performed using R Statistical Software (version 3.6.2; R Foundation for Statistical Computing, Vienna, Austria).

Of the 332 people who died by suicide, 182 (55%) used a firearm (Table 1). Those who died by firearm suicide and non-firearm suicide had similar distributions of age, race, and ethnicity. Similar to extant research (WISQARS 2020), a higher proportion of individuals dying by firearm suicide was male (83.5% vs. 67.3%), have military affiliation (39.0% vs. 19.3%), and were less likely to have a known mental health diagnosis (47.3% vs. 64.7%) compared to those who died from other means (Table 1). A higher proportion of individuals dying by firearm suicide was male (83.5% vs. 67.3%), had military affiliation (39.0% vs. 19.3%). Those dying by firearm suicide was less likely to have a known mental health diagnosis (47.3% vs. 64.7%) compared to those who died from other means (Table 1). Among those who died by firearm suicide, 85.2% used a handgun (Table 1) and 89.0% suffered a gunshot wound to the head/neck (Table 2).

**Table 1:** Demographic information in all suicide decedents in El Paso from 2018 to 2019

	Firearm (*n*(%), *N* = 182)	Non-Firearm (*n*(%), *N* = 150)
Age at death, in years Mean (SD)	42.4 (19.5)	41.4 (15.8)
Gender (male)	152 (83.5)	101 (67.3)
Method		
Firearm	182 (100.0)	–
Sharp object	–	3 (2.0)
Blunt object	–	1 (0.7)
Poisoning	–	48 (32.0)
Hanging	–	89 (59.3)
Other	–	6 (4.0)
More than 1	–	2 (1.3)
Unknown	–	1 (0.7)
Military affiliation^a^		
No	109 (59.9)	116 (77.3)
Yes	72 (39.6)	32 (21.3)
Unknown	1 (0.5)	2 (1.3)
Race		
White	157 (86.3)	129 (86.0)
Black	7 (3.8)	6 (4.0)
Asian	3 (1.6)	Suppress
American Indian	Suppress	Suppress
Native Hawaiian or Other Pacific	Suppress	0 (0.0)
Islander	5 (2.7)	4 (2.7)
Other	6 (3.3)	5 (3.3)
Multiracial	Suppress	3 (2.0)
Unknown		
Ethnicity		
Not hispanic	161 (88.5)	127 (84.7)
Hispanic	19 (10.4)	22 (14.7)
Unknown	Suppress	Suppress
Personal Relationship Status		
Currently in relationship^b^	99 (54.4)	61 (40.1)
Not currently in relationship	51 (28.0)	42 (28.0)
Unknown	32 (17.6)	47 (31.3)
Known history of mental health diagnosis^c^		
Yes	86 (47.3)	97 (64.7)
No/unknown	96 (52.7)	53 (35.3)
Type of firearm		
Handgun	–	155 (85.2)
Rifle or shotgun	–	26 (14.3)
Unknown	–	1 (0.6)
Owner of firearm used in suicide		
Decedent	–	136 (74.7)
Family of decedent	–	17 (9.3)
Other	–	8 (4.4)
Unknown	–	21 (11.5)

Data suppressed if cell counts are less than 3

^a^Active service member or veteran

^b^Married, civil union, domestic partnership, or other partner (boyfriend, girlfriend, unspecified intimate partner)

^c^These include depression, anxiety, bipolar disorder, schizophrenia as documented in Medical Examiner records

**Table 2:** Circumstances surrounding suicide decedent, stratified by firearm and non-firearm suicide

	Total (*n*(%), *N* = 332)	Firearm suicide (*n*(%), *N* = 182)	Non-firearm suicide (*n*(%), *N* = 148)
Who discovered the decedent?			
Family/friend^a^	200 (60.2)	107 (58.8)	93 (62.8)
Stranger/acquaintance^b^	70 (21.0)	41 (22.5)	29 (19.5)
First responder^c^	74 (22.4)	44 (24.2)	30 (20.3)
Dead when found	243 (73.2)	121 (66.5)	122 (81.3)
Dying when found	86 (25.9)	58 (31.9)	28 (18.7)
Unknown	3 (1.0)	3 (1.7)	0 (0.0)
Forced witnessing^d^	63 (19.0)	47 (25.8)	16 (10.8)
Location decedent discovered			
Home^e^	237 (71.4)	128 (70.3)	109 (72.7)
Other	93 (28.0)	53 (29.1)	40 (26.7)
Unknown	2 (0.6)	1 (0.6)	1 (0.7)
Location of gun shot wound			
Head/neck	–	162 (89.0)	–
Trunk	–	20 (11.0)	–

Outdoors or in vehicle away from residence, hotel/motel

^a^Includes if any of the following were present: current/former intimate partners, parent, child, sibling, uncle, grandchild, friend, roommate

^b^Includes if any of the following were present: coworker, neighbor, other known to the decedent

^c^Includes only: first responders (police, fire, ambulance) and strangers who are not first responders

^d^Defined as the suicide decedent killing themselves in front of a whiteness

^e^Inside or outside of residence

Most suicide decedents died in their home (71.4%) and were found by a family member or friend of the decedent (60.2%). Of these, 90 (49.5%) were current or former intimate partners, 74 (40.7%) were 1st degree relatives, 39 (21.4%) were other relatives and friends. The remainder were found by a stranger/acquaintance (20.0%) or a first responder (22.4%).

Most (73.2%) suicide decedents were already deceased when found; however, more non-firearm suicide decedents were already deceased when found compared to firearm suicide decedents (81.3% vs. 66.5%). Significantly more firearm suicide events included forced witnessing compared to non-firearm suicide decedents (25.8% vs. 10.3%); (Table 2). Both who discovered the decedent and the decedent's location were similar between firearm and non-firearm suicide events.

## Discussion

In this analysis of 332 suicide decedents in El Paso County, Colorado from 2018 to 2019, over two-thirds of suicide decedents died in their home and more than half were discovered by a family member or friend. The remaining 43% of suicide decedents were discovered by strangers/acquaintances or first responders. Little is known about the bereavement and trauma-related outcomes among people who discover a suicide decedent. Given that first-degree relatives of suicide decedents are at increased risk of suicide themselves, interventions targeting survivor-victims who discover the suicide decedent may be warranted to address trauma and mental health effects (Harvard Womens Health Watch 2009). The disfigurement associated with violent methods of suicide (especially firearm suicide to the head/neck) may be an important factor when understanding these traumatic impacts. Additionally, suicides that involve forced witnessing (e.g. shooting oneself in the midst of an argument with an intimate partner or jumping in front of a moving vehicle) and discovering the victim while still alive and being unable to save them are also important factors to consider in terms of the vicarious trauma for the suicide discoverer. Data included in this report are limited to that collected by the coroner’s office and police reports in suicide death investigations in one county. Though there may be some differences, our comparative data appear similar to other firearm vs. non-firearm suicide data at the national level (WISQARS 2020). Contagion effects of suicide have been studied, particularly among adolescents and the impact of media reports reporting suicides and resulting clusters (Gould and Davidson 1988). Future research may evaluate contagion effects of suicide stratified by who discovers the suicide decedent. Lastly, further studies exploring who discovers suicide decedents and how this impacts their mental health may be important to develop-targeted postvention strategies for supporting impacted family, friends, first responders, and strangers.

## Data Availability

All data are available on request from the El Paso County Coroners Office.
